# Supplementary material for: Investigating the influence of language teachers’ constructivist self-efficacy on their practice of constructivism in Ghanaian language and culture instruction
Source: PLoS One. 2025 Mar 28;20(3):e0320246. doi: 10.1371/journal.pone.0320246 (PMC11952250; doi:10.1371/journal.pone.0320246)
Supplement: Attachment — Submitted filename: Review Comments_PONE-D-24-34525.docx [file pone.0320246.s001.docx]

Title: Investigating the Influence of Teachers’ Constructivist Self-Efficacy on Their Practice of Constructivism in Ghanaian Language Instruction

Manuscript Number: PONE-D-24-34525

Thank you for the opportunity to review the manuscript entitled “Investigating the Influence of Teachers’ Constructivist Self-Efficacy on Their Practice of Constructivism in Ghanaian Language Instruction”. The study addresses a pertinent issue in educational reform, particularly the transition from behaviorist to constructivist instructional philosophies in Ghana. The manuscript is well-structured and presents a mixed-methods approach to understanding how teacher self-efficacy influences the implementation of constructivist teaching practices. Below are my detailed comments and recommendations for the authors to consider.

**Detailed Comments:**

1. **Introduction:** 1) The manuscript presents a clear problem statement, emphasizing the resistance of teachers to educational change and the need to explore teachers’ sense of efficacy in the context of constructivist teaching. However, more specific data or updated references that quantify the extent of teacher resistance or highlight the impact of teacher-centered learning on student outcomes should be provided. 2) While the introduction hints at the study’s practical implications, it should more explicitly address how this research advances the theoretical understanding of constructivist teaching and teacher self-efficacy. What new insights does it offer? Such as theoretical advancements, methodological innovations, or new empirical evidence. 3) The last paragraph of the introduction, which outlines the structure of the paper, is somewhat redundant and less informative. Consider deleting the section that merely lists the content of the forthcoming sections. Instead, conclude the introduction with a compelling summary that emphasizes how the study’s design and methodology are specifically tailored to address the research questions.
2. **Literature Review:** 1) Section 2.1: While the overview is thorough, consider integrating more recent studies to ensure the review is current. Additionally, discuss how these constructs of constructivism have been specifically applied or adapted in the Ghanaian educational context or similar settings. 2) Section 2.2: Please include studies that have specifically examined the relationship between self-efficacy and the implementation of constructivist teaching methods. This will provide a more direct link to the research focus.  Additionally, provide a more detailed classification and elaboration of previous studies, including the measurement methods used and the background of the participants (e.g., college teachers, middle school teachers). This detail will help to identify and articulate the gaps in the existing literature more effectively. 3) Section 2.3: Please end with a brief summary of how the literature review informs the research questions and the study’s hypotheses.
3. **Methodology:** 1) Section 3.1: Lack a rationale for choosing this particular mixed-methods design over other possible designs. Discuss how this approach specifically addresses the research questions or hypotheses. Besides, the research procedure mentioned (Thus, in the context of ……) is too general, please add more details here or put the “research procedure” as an independent section. 2) Consider adding a section on data analysis methods, detailing how the quantitative and qualitative data will be analyzed separately and then integrated in the overall interpretation of results.
4. **Results**: 1) Provide more data about the participants, for example the mean value, SD, etc. 2) Provide more context on how the qualitative data were collected and analyzed. Detail the methods used for data collection (e.g., interviews, focus groups) and the data analysis process. Besides, consider using visual aids, such as tables or figures, to summarize key themes or findings from the qualitative data.
5. **Discussions & Conclusions:** 1) Structurally, please separate “Discussion and Conclusion” into two independent parts, in which discussion should include both limitations (Section 6) and implications (Section 7). 2) Expand on how this study contributes to or diverges from previous research, highlighting its unique contributions. Again, the references used in this part is too old to clarify your novel contributions to this field.

**Recommendation:** Major revision
