# Supplementary material for: Investigating the influence of language teachers’ constructivist self-efficacy on their practice of constructivism in Ghanaian language and culture instruction
Source: PLoS One. 2025 Mar 28;20(3):e0320246. doi: 10.1371/journal.pone.0320246 (PMC11952250; doi:10.1371/journal.pone.0320246)
Supplement: Attachment — Submitted filename: My review and recommendation.docx [file pone.0320246.s003.docx]

Manuscript Number: PONE-D-24-34525R1

Manuscript Title: Investigating the Influence of teachers’ constructivist self-efficacy on their practice of Constructivism in Ghanaian language instruction

The study is beneficial and timely for educational discourse within the Ghanaian context. It seeks to improve the policy and practice in Ghana. However, certain aspects of the paper need to be tightened and reviewed to improve the quality. Reviewer 1 and 2 raised concerns that needs to be addressed.

Methodology

The data analysis of the qualitative data needs further elaboration. The interpreted meaning, synthesis and summary of the analysis for presentation are missing in the sub-section.

The reviewer 2 in number 4 raised an issue on the elaboration on the semi-structured interview format and types of questions that were used to operationalised the various forms of constructivist teaching explored in the study. This has not been addressed by the authors in the study.

Results

The point 2 raised by reviewer 1 is very significant to the quality of the manuscript and the authors are supposed to address that.

The reviewer 2 raised an issue of detailing the qualitative results of the study. This has not been addressed and needs to be given the necessary attention by the authors.

The point 7 raised by reviewer 2 needs to be addressed by the authors.
